# Environmental Filtering Effect Drives the Plant Species Distribution in Alpine Grasslands on the Qinghai‐Tibetan Plateau

**DOI:** 10.1002/ece3.71599

**Published:** 2025-06-17

**Authors:** Yikang Cheng, Ding Li, Nadia I. Maaroufi, Jianling You, Wen Zhou, Wensheng Liu, Danhui Qi, Xiang Liu, Yuguo Wang, Xiaoyun Pan, Wenju Zhang, Ji Yang, Shurong Zhou, Zhiping Song

**Affiliations:** ^1^ State Key Laboratory of Wetland Conservation and Restoration, National Observations and Research Station for Wetland Ecosystems of the Yangtze Estuary, Ministry of Education Key Laboratory for Biodiversity Science and Ecological Engineering, and Institute of eco‐Chongming, School of Life Sciences Fudan University Shanghai China; ^2^ School of Ecology Hainan University Haikou China; ^3^ Department of Soil and Environment Swedish University of Agricultural Sciences Uppsala Sweden; ^4^ Institute of Plant Sciences University of Bern Bern Switzerland; ^5^ College of Life Science and Technology Central South University of Forestry and Technology Changsha China; ^6^ College of Environmental Science and Engineering Southwest Forestry University Kunming China; ^7^ State Key Laboratory of Herbage Improvement and Grassland Agroecosystems, College of Ecology Lanzhou University Lanzhou China

**Keywords:** alpine ecosystem, biodiversity maintenance, community assembly, deterministic process, spatial scale, stochastic process

## Abstract

Exploring community assembly is essential for understanding the mechanisms of biodiversity maintenance and species coexistence. In general, stochastic (e.g., dispersal limitation) and deterministic (e.g., environmental filtering) effects have been identified as the two key processes driving community assembly. However, the relative contributions of these two processes and how they vary across different spatial scales remain poorly understood, especially for the high‐diversity grassland ecosystems on Qinghai‐Tibetan Plateau (QTP), which plays a critical role in global climate regulation. In this study, a total of 27 study sites were established along a north–south transect and a west–east transect across the eastern QTP; the two furthest sites were more than 1000 km apart. We analyzed the taxonomic, functional, and phylogenetic diversity and structure of these communities to elucidate the relative importance of dispersal limitation and environmental filtering effects that shape plant distributions at the regional (i.e., encompassing all sites) and the transect scales. A total of 181 species belonging to 99 genera and 34 families of vascular plants were found across all sample sites. Both at the regional and the transect scale, environmental variables were shown to account for a larger proportion of the variation in species composition than spatial variables. Likewise, the plant species diversity (i.e., taxonomic, functional, and phylogenetic diversity) was also primarily influenced by soil and climatic variables rather than by spatial factors. Specifically, mean annual precipitation, mean annual temperature, and soil total carbon content emerged as critical determinants of plant species diversity at the regional scale, while the mean annual temperature was identified as the most important factor at the transect scale. Our results highlight the significance of environmental filtering, rather than dispersal limitation, in shaping plant community dynamics across various spatial scales within the alpine grassland ecosystem, which has crucial implications for plant conservation and biodiversity maintenance under global change scenarios.

## Graphical abstract

Environmental variables do more contributions to the variation of species composition than spatial variables in the eastern Qinghai‐Tibetan Plateau. The patterns of taxonomic, functional, and phylogenetic diversity of the plant community are primarily influenced by mean annual precipitation, mean annual temperature, and total soil carbon content emerged.

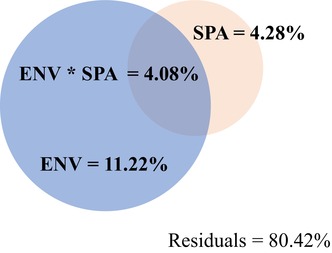

## 1 Introduction

Climate change, such as global warming and altered precipitation regimes, is known to be one of the major environmental filters shaping plant community assemblies, leading to significant alterations to species richness and diversity patterns (Grimm et al. 2013; Pimm et al. 2014; Henn et al. 2024). Understanding the underlying mechanism that drives species diversity patterns in space and time is crucial for determining how plant community dynamics respond to ongoing global climate change (Urban et al. 2016).

From the broadest theoretical viewpoint, it is commonly assumed that large‐scale patterns of species assemblage are generally considered to be driven by two ecological processes, that is, deterministic niche processes (e.g., environmental filtering) and/or neutral processes (e.g., dispersal limitation) (Gravel et al. 2006; Vellend 2010). In recent decades, many studies have endeavored to explicitly test the relative importance of these two kinds of mechanisms in shaping plant community assembly (Kristiansen et al. 2012; Zhang et al. 2018; Shi et al. 2021). However, the driving factors are context‐dependent and no consistent empirical conclusion has yet been established across ecoregions (Myers et al. 2013; Thapliyal et al. 2025) and spatial scales (Carmona et al. 2016). For example, the environmental filtering effect has been identified as the predominant factor influencing community assembly in temperate forest ecosystems, while plant communities appear to exhibit a stronger spatial correlation in tropical regions, which is indicative of the dispersal limitation effect (Myers et al. 2013). Likewise, many studies have found that abiotic filtering effect (result from the elevation and temperature gradients) drives the plant distribution on the Himalayan biodiversity hotspot (Bahukhandi et al. 2024; Sekar et al. 2025). Although there is a growing consensus that community structure is shaped by both niche and neutral processes (Bartlett et al. 2016; Aiello‐Lammens et al. 2017; Shi et al. 2021), the applicability of these mechanisms across different scales remains inadequately explored.

Disentangling the relative influences of deterministic and neutral processes on biodiversity patterns has proven to be challenging because those effects tend to be scale‐dependent (i.e., the size of the study area; Kraft and Ackerly 2010; Chase 2014). Indeed, larger study areas typically increase the size of the species pool and exhibit greater habitat heterogeneity, thereby demonstrating a pronounced environmental filtering effect. Conversely, the dispersal limitation effect may be amplified in smaller areas where there are fewer individuals per unit area and lower environmental heterogeneity (Chase 2014). Besides, divergent patterns of community assembly have also been documented in different ecosystems across various spatial scales (de Bello et al. 2013; Germain et al. 2017; Zhang et al. 2018). For instance, in dry semi‐natural grasslands, abiotic filtering and dispersal limitation dominated the community assembly process at small (i.e., 50 × 50 cm) and large scales (i.e., 0.02–11.63 ha), respectively (de Bello et al. 2013). Similarly, habitat filtering has been identified as the strongest driver at small spatial scales, while dispersal limitation is considered to prevail at relatively larger scales (Zhang et al. 2018). Therefore, it is essential to explicitly consider spatial scale when investigating the processes that govern plant community assembly.

The Qinghai‐Tibet Plateau (QTP) is recognized as the largest and most elevated plateau in the Northern Hemisphere, with an average elevation of 4500 m, and it contains a vast area of alpine grassland ecosystems (Zou et al. 2017). Besides, this region is also a biologically diverse ecosystem supporting high proportions of flora and harboring a large number of protected areas, making it an exemplary platform in which to investigate the relative significance of environmental filtering versus dispersal limitation (Liu et al. 2018), particularly considering the significant global change it is currently experiencing (Henn et al. 2024; Sekar et al. 2025). Indeed, those alpine ecosystems are undergoing more pronounced increases in temperature compared to lower‐elevation areas (Pepin et al. 2022), and are also facing drastic alterations in precipitation patterns (Brunetti et al. 2006). Therefore, the alpine grassland communities on QTP provided a unique backdrop to elucidate the specific drivers of plant community distribution across various spatial scales.

In general, abiotic factors are known to play a critical role in determining species co‐occurrence in extreme environmental conditions, such as the cold alpine grassland ecosystem, while biotic competition effect has been identified as the significant driver of species assemblage in warmer and more humid environments such as rainforests (Louthan et al. 2015). In this study, we conducted a large‐scale investigation (i.e., two transects spanning hundreds to thousands of kilometers) and employed a multi‐faceted approach, including taxonomic, functional, and phylogenetic dimensions, to examine variations in plant diversity and composition across both environmental gradients and spatial scales, specifically at the regional and transect levels. We aimed to test two hypotheses: (i) both environmental filtering and dispersal limitation effects play an important role in shaping plant species diversity at both the regional and the transect scales, and (ii) environmental variables, including climatic and soil characteristics, and spatial factors can best explain the observed changes in plant community composition across these different scales.

## 2 Methods

### 2.1 Study Area

**FIGURE 1:**
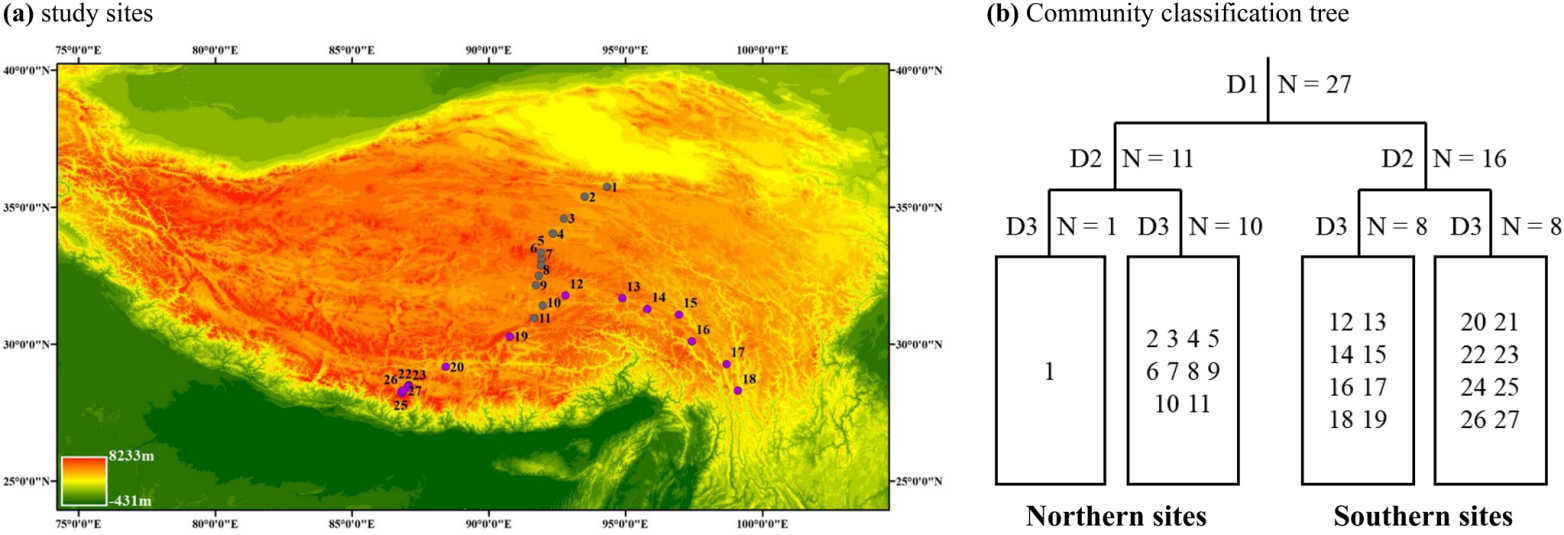
(a) Distribution of study sites in the Qinghai‐Tibet Plateau; (b) Hierarchy and classification tree of the grassland communities in the Qinghai‐Tibet Plateau using two‐way indicator species analysis.

We established 27 study sites along a north‐to‐south transect (N 28°–35° in latitude) and a west‐to‐east transect (E 86°–97° in longitude) spanning the Qinghai‐Tibet Plateau (QTP) (the two furthest sites are more than 1000 km apart) (Figure 1a; Table S1). Our survey sites exhibited an obvious climate gradient, with mean annual temperatures ranging from −5.3°C to 5.0°C and mean annual precipitation varying from 185 to 691 mm. Those sites were selected with similar terrain and the smallest slope, and were located at least 2 km away from anthropogenic disturbances, such as highways. The plant community along the north–south transect was dominated by several graminoids from the genera *Poa*, *Kobresia*, *Carex*, and *Stipa*, as well as various dicots from the genera *Saussurea*, *Pedicularis*, *Potentilla*, and *Gentiana*. In contrast, the primary vegetation types gradually transitioned from alpine steppe to alpine meadow along the west–east transect.

### 2.2 Sample Collection

Field sampling was conducted during the peak of the growing season at the end of August 2018. Specifically, we established a 30 × 30 m plot at each site, within which ten 1 × 1 m subplots were systematically arranged along the diagonal of the main plot, maintaining a 3 m interval between adjacent subplots. The central and terminal subplots along the diagonal were utilized to assess community composition and quantify species abundance, resulting in 81 (i.e., 27 sites * 3 replicates) subplots in total. For the plant community, we inventoried all plant species rooted within the plots and then recorded species identity and abundance by morphological identification. A total of 181 species belonging to 99 genera and 34 families of vascular plants were found across all sample sites, and many endemic and threatened plant species were recorded within sample sites, such as *Saussurea gossypiphora*, *Meconopsis horridula*, *Gentiana tibetica*, and *Gentiana futtereri* (Table S2).

To measure the phylogenetic relationships among plant species in our study sites, we collected fresh plant leaves of those species, desiccated them using silica gel, and stored them at −20°C until DNA extraction could be performed.

At each site, we also collected three soil cores (10 cm in depth) within each of the three subplots and then mixed the soil cores to obtain a single composite sample per subplot. Soil factor measurements included soil total carbon content (STC), total nitrogen content (STN) and available phosphorus content (SAP). STC and STN were measured using an auto elemental analyzer (ICS1100, Thermofisher), while SAP was measured using the molybdate colorimetric test (Olsen method).

### 2.3 Functional Trait Measurements

Following the standardized protocols outlined by Cornelissen et al. (2003), we measured six plant functional traits: plant height (H, cm), specific leaf area (SLA, cm^2^/g), plant biomass (g), leaf total nitrogen content (LN, mg/g), leaf total carbon content (LC, mg/g), leaf available phosphorus content (LP, mg/g) and leaf carbon/nitrogen ratio (LCN). Within the three subplots located at the center and at either end of the diagonal line, we selected 10–20 individuals of each plant species present and measured their heights. Several of the plant species in the three subplots had very few individuals; in such cases, we sampled other individuals from the other subplots to meet the minimum number of plant individuals (> 10). For each individuals, we also selected three mature and undamaged leaves to measure leaf area (using the LA‐S Leaf Area Analysis software, Wseen Detection Technology Co. Ltd.), and then we dried the leaves at 65°C to a constant mass and weighed them to the nearest 0.0001 g to calculate the specific leaf area (SLA). The leaf carbon and nitrogen contents were measured using an auto elemental analyzer (ICS1100, Thermo Fisher), and the total phosphorus concentration was determined using persulfate oxidation followed by the acid molybdate method.

### 2.4 Climate Data

Two climate variables (i.e., annual mean temperature (MAT) and annual mean precipitation (MAP)), which have been shown to significantly influence plant communities on the QTP (Zhang et al. 2024), were obtained for the 27 sample sites from the website Worldclimate (www.worldclim.org). Briefly, temperature and precipitation are averaged over 30 years (1970–2000) of historical data with a resolution of 30 s. Finally, we also recorded the longitude and latitude of each plot using a handheld GPS device.

### 2.5 Community Phylogeny

The methods for DNA extraction, amplification and sequencing followed the protocol provided by Kress et al. (2010). Briefly, three sequences (i.e., two chloroplast gene sequences (MatK and rbcL) and one nuclear gene sequence (ITS)) of each species were aligned by MUSCLE (version 5) separately and then concatenated together to construct an entire matrix. We estimated a maximum‐likelihood phylogeny using RAxML with 1000 bootstrap replicates and then used a semi‐parametric rate‐smoothing method to transform the phylogeny into an ultrametric evolutionary tree using the ‘*chrono*’ function in R package ‘*ape*’ (version 5.0; Paradis and Schliep 2019). Likewise, considering that evolutionary trees constructed based on DNA sequences may be biased in the classification of species below the family level, we also generated a phylogenetic tree using the ‘*V.PhyloMaker2’* package (Jin and Qian 2022) for comparison.

### 2.6 Community Diversity and Structure

Three diversity components of the plant community were assessed for each plot: taxonomic, functional, and phylogenetic diversity. Briefly, we defined taxonomic and phylogenetic diversity as taxonomic richness and as Faith's PD (Faith 1992), respectively. For functional diversity, we measured the functional richness and calculated the community‐weighted mean values (CWM, Garnier et al. 2004) of H, SLA, LC, LN, LP, and LCN, weighted by the species abundance for each plot.

To characterize the community functional and phylogenetic structures, we first calculated the abundance‐weighted mean functional trait distance (MFD) and abundance‐weighted mean pairwise phylogenetic distance (MPD) (Webb et al. 2002). Then, we measured the standardized effect size (SES) of the MFD and MPD based on null distributions by shuffling species labels in the phylogeny and the trait matrix 999 times. Then, we calculated the SES values of MPD and MFD as follows.

$$\mathrm{SES}.\mathrm{MFD}\;\left(\mathrm{SES}.\mathrm{MPD}\right)=\left(\chi_{\mathrm{obs}}–\chi_{\text{null}}\right)/{\mathrm{SD}}_{\text{null}}$$

Where *χ*_obs_ is the observed value, *χ*_null_ is the mean, and SD_*null*_ is the standard deviation of the simulated values. Positive values of SES.MFD and/or SES.MPD indicates functional and/or phylogenetic over‐dispersion, while negative values indicate functional and/or phylogenetic clustering, respectively. All the functional and phylogenetic analyses were performed in R 4.4.1 (R Core Team, 2024) with the ‘*FD*’ package (version 1.0–12.3; Laliberté and Legendre 2010) and the ‘*picante*’ package (version 1.8.2; Kembel et al. 2010). Notably, the Faith's PD and SES.MPD calculated based on the two phylogenetic trees was highly correlated (*r* > 0.80; Figure S1).

### 2.7 Statistical Analyses

Due to the difference of vegetation composition and climatic context, we applied two‐way indicator species analysis (TWINSPAN) to classify these 27 sites based on the community composition, and all sites could be classified into two groups, either the southern or the northern vegetation (Figure 1b). Thus, all the following analyses were performed both at the regional scale (i.e., all sites) and the transect scale (i.e., northern and southern sites).

#### 2.7.1 Effect of Abiotic Variables on Plant Diversity and Structure

Because STN was highly correlated with STC (*r* > 0.80; Figure S2), we removed TN and used the C:N ratio (SCN) instead. Then linear mixed effect (LME) models were applied to test the effects of spatial (i.e., longitude and latitude) and environmental (i.e., MAP, MAT, STC, SAP and SCN) variables on plant diversities (i.e., taxonomic richness, functional richness, Faith's PD and CWM of plant traits) and structure (i.e., SES.MFD and SES.MPD), with the abiotic variable as the fixed effect and ‘site’ as a random effect (model sequence: ‘∼ individual abiotic variable + (1|site)’). LME models were fitted using the ‘*lmer()*’ function in the ‘*lme4*’ package (version 1.1–35; Bates et al. 2015). Response variables were log‐transformed for normality.

#### 2.7.2 Relative Importance of Environmental and Spatial Variables

The LME models were applied to test the explanatory effects of the environmental and spatial variables on the community diversities and structure using the ‘*lmer()*’ function in the ‘*lme4*’ package (version 1.1–35; Bates et al. 2015). The LME included all environmental (i.e., MAP, MAT, STC, SAP and SCN) and spatial variables (i.e., longitude and latitude) as fixed effects and ‘*site*’ as a random effect. To compare the explanatory power of the environmental and spatial variables after the LME analysis, we then applied a variance partitioning analysis using the ‘*glmm.hp*’ function in the ‘*glmm.hp*’ package (version 0.1–7; Lai et al. 2022). Response variables were log‐transformed for normality.

Lastly, we also applied variance partitioning analysis to partition the effect of environmental (i.e., MAP, MAT, STC, SAP and SCN) and spatial parameters (i.e., longitude and latitude) on plant community composition using the ‘*varpart()*’ function in the ‘*vegan*’ package (version 2.6–10; Oksanen et al. 2019).

## 3 Results

### 3.1 Effect of Abiotic Variables on Plant Diversity and Structure

At the regional scale (i.e., all sites) and transect scale (i.e., southern sites), the results of the linear mixed‐effects model (LME) indicate that the plot‐level taxonomic, functional, and phylogenetic diversities of the plant community were significantly related to longitude, mean annual precipitation (MAP), and soil total carbon content (STC) (Table 1; Table S3). However, for the plant communities at the northern sites, only longitude had a significant effect on the taxonomic richness of the northern sites, and all other spatial and environmental variables had negligible effects on functional and phylogenetic diversities (Table S3).

**TABLE 1:** Effects of abiotic variables on plot‐level plant diversities (i.e., taxonomic, functional, and Faith's PD) at the regional scale (i.e., all sites).

Variables	Taxonomic richness		Functional richness		Faith's PD	
	*p*	*R* ^2^m	*p*	*R* ^2^m	*p*	*R* ^2^m
Longitude	**0.004**	**0.239**	**0.001**	**0.234**	**0.012**	**0.186**
Latitude	0.491	0.016	0.683	0.005	0.930	0.001
MAP	**0.021**	**0.160**	**0.003**	**0.199**	**0.015**	**0.176**
MAT	0.256	0.043	0.730	0.003	0.701	0.005
SAP	0.581	0.010	0.787	0.002	0.787	0.002
STC	**0.008**	**0.207**	**0.018**	**0.136**	**0.025**	**0.150**
SCN	0.680	0.006	0.796	0.002	0.727	0.004

*Note:* The corresponding *p*‐value and marginal *R* ^*2*^m (variance explained by the fixed effects only) are displayed. Response variables were log‐transformed for normality. Site was treated as a random effect. Bold values indicate significant relationship (*p* < 0.05).

Abbreviations: MAP, mean annual temperature; MAT, mean annual precipitation; SAP, soil available phosphorus content; SCN, soil carbon/nitrogen ratio; STC, soil total carbon content.

We also found that various variables could explain the changes in the community‐weighted mean (CWM) values of plant traits, as well as of the functional and phylogenetic structures across different scales (Tables S4 and S5). For example, for all sites, MAP had a significant effect on CWM of leaf carbon content, leaf carbon/nitrogen ratio and specific leaf area; while CWM of leaf carbon/nitrogen ratio was significantly affected by mean annual temperature (MAT).

### 3.2 Relative Importance of Environmental and Spatial Variables

**FIGURE 2:**
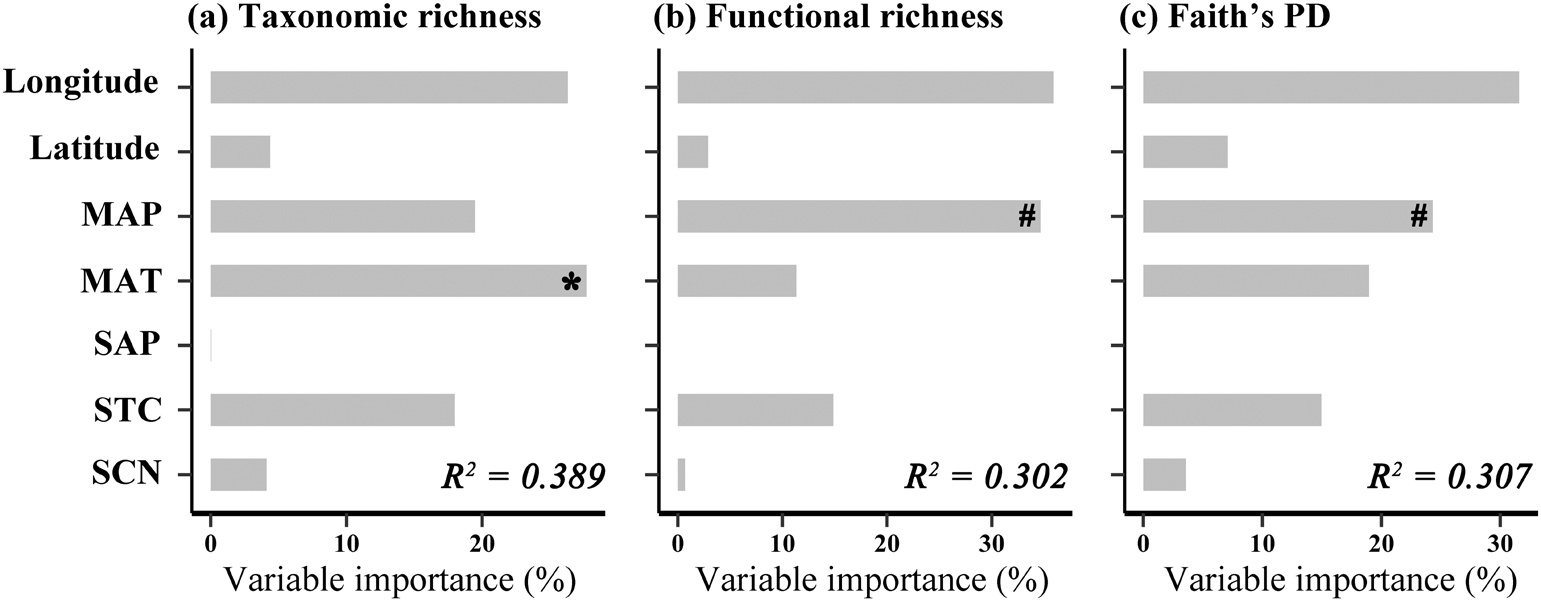
Fraction of the variation in plant taxonomic (a), functional (b), and phylogenetic (c) diversities explained by spatial and environmental predictors at the regional scale. Response variables were log‐transformed for normality. Significance: ^#^ *p* < 0.1, *p < 0.05. MAP, mean annual temperature; MAT, mean annual precipitation; SAP, soil available phosphorus content; SCN, soil carbon/nitrogen ratio; STC, soil total carbon content.

**FIGURE 3:**
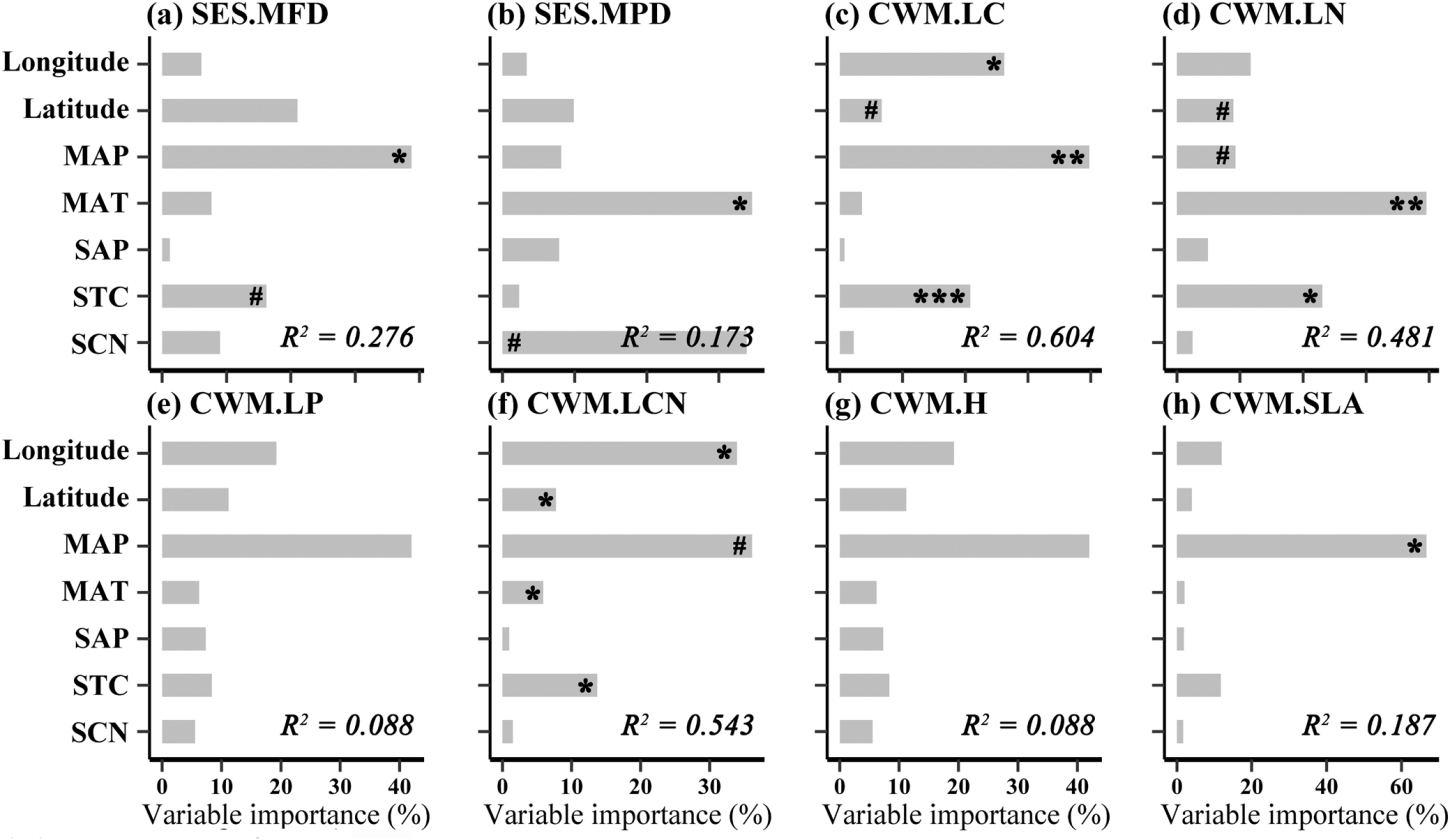
Fraction of variation in plant community structure and the weighted mean values of plant traits explained by spatial and environmental predictors at the regional scale (i.e., all sites). Significance: ^#^ *p* < 0.1, *p < 0.05, **p < 0.01, ***p < 0.001. CWM.H, community weighted mean of plant height; CWM.LC, community weighted mean of leaf carbon content; CWM.LCN, community weighted mean of leaf carbon/nitrogen ratio; CWM.LN, Community weighted mean of leaf nitrogen content; CWM.LP, community weighted mean of leaf available phosphorus content; CWM.SLA, community weighted mean of specific leaf area; MAP, mean annual temperature; MAT, mean annual precipitation; SAP, soil available phosphorus content; SCN, soil carbon/nitrogen ratio; SES.MFD, standardized effect size of mean functional trait distance; SES.MPD, standardized effect size of mean pairwise phylogenetic distance; STC, soil total carbon content.

**FIGURE 4:**
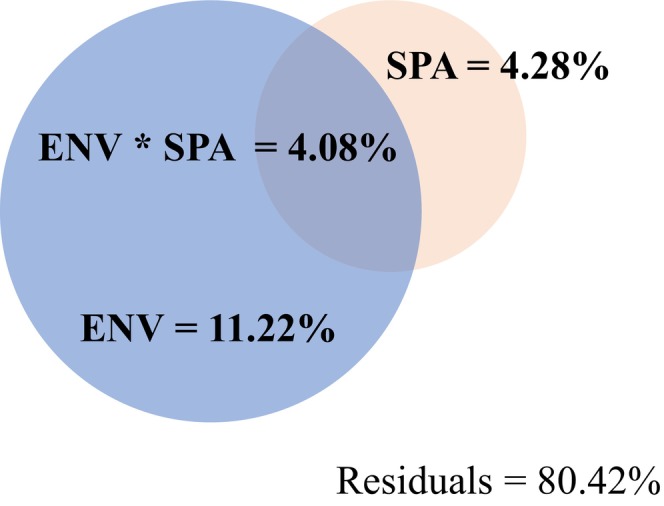
The fraction of the variation in species composition explained by the spatial (i.e., SPAT) and environmental (i.e., ENV) variables, and their interaction (i.e., ENV * SPA) at the regional scale (i.e., all sites).

Based on the variation partitioning analysis (VPA), we found that all candidate variables accounted for 30.2%–38.9% of the variation in plant taxonomic, functional, and phylogenetic diversities at the regional scale. MAT and MAP were identified as two important predictors (Figure 2). At the transect scale, the VPA results indicated that MAT was the most important predictor for the phylogenetic diversity of northern sites, as well as for the taxonomic and phylogenetic diversity of southern sites (Figure S3). Likewise, we found similar results based on the phylogenetic tree constructed by V.PhyloMaker (Figure S4). Environmental variables were also found to be more important than spatial variables for the community‐weighted means (CWM) of plant traits, functional structure, and phylogenetic structure at both the regional (Figure 3) and the transect (Figure S5) scales. Likewise, for the community composition, we observed that environmental variables could explain more variation than spatial variables at both the regional (Figure 4) and the transect (Figure S6) scales.

## 4 Discussion

Through a large‐scale sampling of alpine grasslands across the Qinghai‐Tibet Plateau (QTP), this study aimed to assess the relative importance of abiotic filtering and dispersal limitation at various spatial scales by integrating a comprehensive analysis of taxonomic, functional, and phylogenetic diversities. In general, our findings indicate that climatic variables and soil factors, rather than spatial variables, better explain the observed variation in taxonomic, functional, and phylogenetic community diversity and structure, which provided compelling evidence that environmental filtering predominantly influences the community assembly process in alpine grasslands and that dispersal limitation plays a relatively minor role, regardless of the regional or transect scale. Overall, this study found strong support for the environmental filtering effect to be a key driver of alpine plant community dynamics.

At both the regional and the transect scales, abiotic factors, including mean annual temperature (MAT) and soil total carbon content (STC), accounted for a greater proportion of variation in taxonomic, functional, and phylogenetic diversity than spatial variables (see Figure 2; Figure 3; Figures S3 and S5), which is partly consistent with a previous study conducted in this region (Sekar et al. 2023). This finding underscores the predominant influence of environmental filtering on the assembly of alpine grassland communities. Indeed, many previous studies have also reported similar patterns in alpine ecosystems (Chalmandrier et al. 2017; Yang et al. 2022). For instance, mean annual precipitation (MAP) emerged as a significant explanatory variable for shifts in community structure, as documented for vascular plants across global, regional, and local scales (Dufour et al. 2006; Kreft and Jetz 2007; Crous et al. 2013). Notably, our results indicate that the functional (i.e., standardized effect size of mean functional trait distance, SES.MFD) and phylogenetic structure (standardized effect size of mean pairwise phylogenetic distance, SES.MPD) of northern and southern communities were influenced by different abiotic factors (Figure S5). This variation may be attributed to the significant differences in both vegetation type and environmental conditions between the two transects (Figure 1).

Consistent with our hypothesis, environmental variables were found to account for a greater proportion of the variation in community composition than spatial factors at both the regional and the transect scales (Figure 4; Figure S6). However, the disparity between the explanatory power of environmental and spatial factors was less pronounced at the regional scale than at the transect scale (Figure 2). This observation corroborates previous research indicating that dispersal limitation effects may account for greater variation in species composition at larger scales than at smaller scales (de Bello et al. 2013; Talbot et al. 2014; Germain et al. 2017; Zhang et al. 2018). In contrast, some studies have reported that community assembly is more significantly influenced by dispersal limitation at smaller scales, while abiotic filtering exerts a stronger influence at larger scales, particularly in tropical rainforest ecosystems (Punchi‐Manage et al. 2013). One potential explanation is that this study employed larger‐scale sampling compared with previous studies, thus resulting in greater habitat heterogeneity, even at relatively small scales (i.e., transect scale). Mechanically, greater environmental heterogeneity may favor the establishment of the most suitable plants from a given species pool under the specific conditions (Chase 2014; Leibold et al. 2022). Additionally, another possible explanation is that, despite our efforts to incorporate significant climate and soil nutrient variables, the impact of dispersal limitation may have been overestimated due to the omission of certain abiotic variables that could potentially influence plant species distribution (Legendre et al. 2009; Siefert et al. 2013).

Undeniably, the variance partitioning analysis demonstrated that spatial factors could account for a fraction of the variation in community composition (Figure 2), which suggested that neutral processes (i.e., dispersal limitation) play a certain role in the plant assembly of alpine grassland ecosystems. Indeed, plant species exhibit considerable variability in their dispersal ability, which thereby results in a significant dispersal limitation effect (Stein et al. 2008). Furthermore, it should be noted that increasing geographic distances can lead to changes in environmental conditions across different sites (Gilbert and Lechowicz 2004). The spatial effects observed in community assembly might be associated with environmental variability through species‐habitat relationships (Legendre et al. 2009). Consequently, the significant correlation between spatial variables and community composition may also be influenced by ecological filtering processes.

## 5 Conclusions

In utilizing taxonomic, functional, and phylogenetic methodologies across various spatial scales, this study demonstrated that environmental variables account for a greater proportion of the variation in taxonomic, functional, and phylogenetic diversity, as well as in community composition, than do spatial predictors, which provided robust experimental support that deterministic processes underlie community assembly, and not neutral ones. Overall, this study not only offered novel insights into the community assembly dynamics of alpine grassland ecosystems but also underscores the necessity for a more nuanced understanding of how plant communities respond to global climate change. Additionally, while our study primarily focused on above‐ground functional traits, future research should expand to incorporate below‐ground plant root traits and root‐associated microbial communities further to enhance a comprehensive understanding of plant community assembly processes (Laughlin 2014; Bai et al. 2022). Furthermore, by revealing the relative importance of environmental factors on biodiversity patterns, this study provides a causal roadmap for grassland management and a basis for managers to optimize interventions to improve resilience and sustainability.

## Author Contributions

**Yikang Cheng:** formal analysis (lead), methodology (equal), writing – original draft (equal), writing – review and editing (equal). **Ding Li:** data curation (equal), formal analysis (equal), methodology (equal), software (equal), writing – original draft (equal), writing – review and editing (equal). **Nadia I. Maaroufi:** supervision (equal), writing – review and editing (equal). **Jianling You:** investigation (equal), methodology (equal). **Wen Zhou:** investigation (equal), methodology (equal). **Wensheng Liu:** conceptualization (equal), investigation (equal), methodology (equal). **Danhui Qi:** investigation (equal), methodology (equal). **Xiang Liu:** methodology (equal), supervision (equal). **Yuguo Wang:** conceptualization (equal), investigation (equal). **Xiaoyun Pan:** methodology (equal), supervision (equal). **Wenju Zhang:** supervision (equal). **Ji Yang:** supervision (equal). **Shurong Zhou:** supervision (equal). **Zhiping Song:** conceptualization (lead), funding acquisition (lead), investigation (lead), resources (lead), writing – review and editing (equal).

## Ethics Statement

This work did not require ethical approval and work permission.

## Conflicts of Interest

The authors declare no conflicts of interest.

## Supporting information


Data S1.

## Data Availability

All data is available in the Dryad Digital Repository: http://dx.doi.org/10.5061/dryad.zkh1893gp.
