# Supplementary material for: Environmental Filtering Effect Drives the Plant Species Distribution in Alpine Grasslands on the Qinghai‐Tibetan Plateau
Source: Ecol Evol. 2025 Jun 17;15(6):e71599. doi: 10.1002/ece3.71599 (PMC12171928; doi:10.1002/ece3.71599)

**Environmental filtering effect drives the plant species distribution in alpine grasslands on the Qinghai-Tibetan Plateau**

Yikang Cheng^1, 2^**†**, Ding Li^1^**†**, Nadia I. Maaroufi^3, 4^, Jianling You^1^, Wen Zhou^1^, Wensheng Liu^1, 5^, Danhui Qi^6^, Xiang Liu^7^, Yuguo Wang^1^, Xiaoyun Pan^1^, Wenju Zhang^1^, Ji Yang^1^, Shurong Zhou^2^, Zhiping Song^1*^

*^1^ Ministry of Education Key Laboratory for Biodiversity Science and Ecological Engineering, Coastal Ecosystems Research Station of the Yangtze River Estuary, Institute of Biodiversity Science, School of Life Sciences, Fudan University, 2005 Songhu Road, Shanghai 200438, P. R. China*

*^2^ School of Ecology, Hainan University, Haikou 570228, P. R. China*

*^3^* *Department of Soil and Environment, Swedish University of Agricultural Sciences, 75007 Uppsala, Sweden*

*^4^* *Institute of Plant Sciences, University of Bern, 3013 Bern, Switzerland*

*^5^ College of Life Science and Technology, Central South University of Forestry and Technology, Changsha, 410018, China*

*^6^ College of Environmental Science and Engineering, Southwest Forestry University, Kunming, 650224, China*

^7^ *State Key Laboratory of Herbage Improvement and Grassland Agroecosystems, College of Ecology, Lanzhou University, 222 Tianshui South Road, Lanzhou, 730000, P. R. China*

**ORCID information:**

Yikang Cheng: [https://orcid.org/0000-0003-4942-3904](https://orcid.org/0000-0003-4942-3904%20)

Zhiping Song: https://orcid.org/0000-0001-8029-3787

**†**These authors contributed equally to this work.

***Corresponding author:** Zhiping Song, E-mail: songzp@fudan.edu.cn

**Table S1.** Information of sample sites.

| **Site** | **Longitude** | **Latitude** | **Elevation** | **MAT** | **MAP** |
| --- | --- | --- | --- | --- | --- |
| site1 | 94.31717 | 35.7501 | 4141 | -4.2 | 185 |
| site2 | 93.49838 | 35.38416 | 4501 | -4.23333 | 229 |
| site3 | 92.74425 | 34.59175 | 4667 | -5.30417 | 285 |
| site4 | 92.34186 | 34.04006 | 4706 | -4.0875 | 296 |
| site5 | 91.90418 | 33.34674 | 4798 | -5.25833 | 317 |
| site6 | 91.91141 | 33.07893 | 4940 | -5.17917 | 331 |
| site7 | 91.91796 | 32.8718 | 5156 | -5.125 | 288 |
| site8 | 91.82516 | 32.4923 | 4907 | -4.02917 | 354 |
| site9 | 91.7209 | 32.14981 | 4809 | -3.25 | 368 |
| site10 | 91.96232 | 31.40508 | 4525 | -3.64167 | 367 |
| site11 | 91.65466 | 30.95407 | 4766 | -1.2125 | 343 |
| site12 | 92.79242 | 31.77885 | 4279 | -0.3875 | 458 |
| site13 | 94.87535 | 31.67567 | 4450 | -1.5625 | 538 |
| site14 | 95.79206 | 31.28838 | 3695 | -1.53333 | 541 |
| site15 | 96.94859 | 31.08024 | 4601 | -0.99167 | 526 |
| site16 | 97.4118 | 30.10319 | 4083 | 1.45 | 508 |
| site17 | 98.68619 | 29.27585 | 4224 | 2.954167 | 552 |
| site18 | 99.10172 | 28.30963 | 4323 | 2.1 | 691 |
| site19 | 90.759 | 30.28335 | 4691 | 2 | 316 |
| site20 | 88.4224 | 29.18186 | 4918 | 5.091667 | 396 |
| site21 | 87.06886 | 28.50325 | 5134 | -1.60833 | 262 |
| site22 | 87.06347 | 28.49314 | 4863 | 0.075 | 287 |
| site23 | 87.04165 | 28.47789 | 4534 | 1.179167 | 308 |
| site24 | 87.02022 | 28.42711 | 4185 | 2.245833 | 362 |
| site25 | 86.82064 | 28.21053 | 4932 | -2.82083 | 311 |
| site26 | 86.81028 | 28.27956 | 4729 | -1.23333 | 324 |
| site27 | 86.87189 | 28.31119 | 4558 | 0.258333 | 342 |

MAP: Mean annual temperature; MAT: Mean annual precipitation.

**Table S2.** Complete species list of all species present at the sample sites.

| ***Species*** | ***Genus*** | **Family** |
| --- | --- | --- |
| *Chenopodium prostratum* | *Chenopodium* | Amaranthaceae |
| *Salsola monoptera* | *Salsola* | Amaranthaceae |
| *Allium cyaneum* | *Allium* | Amaryllidaceae |
| *Allium fasciculatum* | *Allium* | Amaryllidaceae |
| *Allium przewalskianum* | *Allium* | Amaryllidaceae |
| *Allium spicatum* | *Allium* | Amaryllidaceae |
| *Chamaesium paradoxum* | *Chamaesium* | Apiaceae |
| *Heracleum millefolium* | *Heracleum* | Apiaceae |
| *Heracleum nepalense* | *Heracleum* | Apiaceae |
| *Ligusticum daucoides* | *Ligusticum* | Apiaceae |
| *Ajania khartensis* | *Ajania* | Asteraceae |
| *Ajania tenuifolia* | *Ajania* | Asteraceae |
| *Anaphalis pannosa* | *Anaphalis* | Asteraceae |
| *Artemisia annua* | *Artemisia* | Asteraceae |
| *Artemisia nanschanica* | *Artemisia* | Asteraceae |
| *Artemisia sieversiana* | *Artemisia* | Asteraceae |
| *Artemisia stricta* | *Artemisia* | Asteraceae |
| *Artemisia vestita* | *Artemisia* | Asteraceae |
| *Artemisia vexans* | *Artemisia* | Asteraceae |
| *Artemisia wellbyi* | *Artemisia* | Asteraceae |
| *Aster alpinus* | *Aster* | Asteraceae |
| *Aster flaccidus* | *Aster* | Asteraceae |
| *Cremanthodium ellisii* | *Cremanthodium* | Asteraceae |
| *Leibnitzia nepalensis* | *Leibnitzia* | Asteraceae |
| *Leontopodium himalayanum* | *Leontopodium* | Asteraceae |
| *Leontopodium nanum* | *Leontopodium* | Asteraceae |
| *Leontopodium ochroleucum* | *Leontopodium* | Asteraceae |
| *Pseudoyoungia gracilipes* | *Pseudoyoungia* | Asteraceae |
| *Saussurea ceterach* | *Saussurea* | Asteraceae |
| *Saussurea gossypiphora* | *Saussurea* | Asteraceae |
| *Saussurea pachyneura* | *Saussurea* | Asteraceae |
| *Saussurea pumila* | *Saussurea* | Asteraceae |
| *Saussurea subulisquama* | *Saussurea* | Asteraceae |
| *Saussurea taraxacifolia* | *Saussurea* | Asteraceae |
| *Saussurea wellbyi* | *Saussurea* | Asteraceae |
| *Taraxacum maurocarpum* | *Taraxacum* | Asteraceae |
| *Taraxacum sikkimense* | *Taraxacum* | Asteraceae |
| *Taraxacum tibetanum* | *Taraxacum* | Asteraceae |
| *Incarvillea younghusbandii* | *Incarvillea* | Bignoniaceae |
| *Chionocharis hookeri* | *Chionocharis* | Boraginaceae |
| *Lasiocaryum densiflorum* | *Lasiocaryum* | Boraginaceae |
| *Microula tibetica* | *Microula* | Boraginaceae |
| *Braya humilis* | *Braya* | Brassicaceae |
| *Dontostemon glandulosus* | *Dontostemon* | Brassicaceae |
| *Dontostemon pinnatifidus* | *Dontostemon* | Brassicaceae |
| *Draba altaica* | *Draba* | Brassicaceae |
| *Draba glomerata* | *Draba* | Brassicaceae |
| *Draba zangbeiensis* | *Draba* | Brassicaceae |
| *Erysimum diffusum* | *Erysimum* | Brassicaceae |
| *Neotorularia brachycarpa* | *Neotorularia* | Brassicaceae |
| *Cyananthus macrocalyx* | *Cyananthus* | Campanulaceae |
| *Morina longifolia* | *Morina* | Caprifoliaceae |
| *Arenaria bryophylla* | *Arenaria* | Caryophyllaceae |
| *Silene aprica* | *Silene* | Caryophyllaceae |
| *Rhodiola bupleuroides* | *Rhodiola* | Crassulaceae |
| *Rhodiola fastigiata* | *Rhodiola* | Crassulaceae |
| *Rhodiola smithii* | *Rhodiola* | Crassulaceae |
| *Sedum przewalskii* | *Sedum* | Crassulaceae |
| *Sedum wangii* | *Sedum* | Crassulaceae |
| *Blysmus sinocompressus* | *Blysmus* | Cyperaceae |
| *Carex atrofusca* | *Carex* | Cyperaceae |
| *Carex duriuscula* | *Carex* | Cyperaceae |
| *Carex microglochin* | *Carex* | Cyperaceae |
| *Carex moorcroftii* | *Carex* | Cyperaceae |
| *Carex nivalis* | *Carex* | Cyperaceae |
| *Carex parva* | *Carex* | Cyperaceae |
| *Carex pseudofoetida* | *Carex* | Cyperaceae |
| *Kobresia fragilis* | *Kobresia* | Cyperaceae |
| *Kobresia humilis* | *Kobresia* | Cyperaceae |
| *Kobresia myosuroides* | *Kobresia* | Cyperaceae |
| *Kobresia pygmaea* | *Kobresia* | Cyperaceae |
| *Kobresia tibetica* | *Kobresia* | Cyperaceae |
| *Hippophae neurocarpa* | *Hippophae* | Elaeagnaceae |
| *Hippophae tibetana* | *Hippophae* | Elaeagnaceae |
| *Euphorbia altotibetica* | *Euphorbia* | Euphorbiaceae |
| *Euphorbia helioscopia* | *Euphorbia* | Euphorbiaceae |
| *Euphorbia stracheyi* | *Euphorbia* | Euphorbiaceae |
| *Astragalus hendersonii* | *Astragalus* | Fabaceae |
| *Astragalus lasaensis* | *Astragalus* | Fabaceae |
| *Astragalus laxmannii* | *Astragalus* | Fabaceae |
| *Astragalus melilotoides* | *Astragalus* | Fabaceae |
| *Astragalus polycladus* | *Astragalus* | Fabaceae |
| *Astragalus tanguticus* | *Astragalus* | Fabaceae |
| *Hedysarum sikkimense* | *Hedysarum* | Fabaceae |
| *Medicago ruthenica* | *Medicago* | Fabaceae |
| *Oxytropis avisoides* | *Oxytropis* | Fabaceae |
| *Oxytropis deflexa* | *Oxytropis* | Fabaceae |
| *Oxytropis melanocalyx* | *Oxytropis* | Fabaceae |
| *Oxytropis oxyphylla* | *Oxytropis* | Fabaceae |
| *Oxytropis savellanica* | *Oxytropis* | Fabaceae |
| *Sophora moorcroftiana* | *Sophora* | Fabaceae |
| *Tibetia tongolensis* | *Tibetia* | Fabaceae |
| *Tibetia yunnanensis* | *Tibetia* | Fabaceae |
| *Comastoma falcatum* | *Comastoma* | Gentianaceae |
| *Gentiana alsinoides* | *Gentiana* | Gentianaceae |
| *Gentiana aquatica* | *Gentiana* | Gentianaceae |
| *Gentiana depressa* | *Gentiana* | Gentianaceae |
| *Gentiana futtereri* | *Gentiana* | Gentianaceae |
| *Gentiana leucomelaena* | *Gentiana* | Gentianaceae |
| *Gentiana prostrata* | *Gentiana* | Gentianaceae |
| *Gentiana pseudosquarrosa* | *Gentiana* | Gentianaceae |
| *Gentiana straminea* | *Gentiana* | Gentianaceae |
| *Gentiana szechenyii* | *Gentiana* | Gentianaceae |
| *Gentiana tibetica* | *Gentiana* | Gentianaceae |
| *Halenia elliptica* | *Halenia* | Gentianaceae |
| *Lomatogonium brachyantherum* | *Lomatogonium* | Gentianaceae |
| *Swertia alba* | *Swertia* | Gentianaceae |
| *Swertia hispidicalyx* | *Swertia* | Gentianaceae |
| *Iris goniocarpa* | *Iris* | Iridaceae |
| *Iris lactea* | *Iris* | Iridaceae |
| *Phlomoides rotata* | *Phlomoides* | Lamiaceae |
| *Salvia trijuga* | *Salvia* | Lamiaceae |
| *Gagea serotina* | *Gagea* | Liliaceae |
| *Linum pallescens* | *Linum* | Linaceae |
| *Euphrasia regelii* | *Euphrasia* | Orobanchaceae |
| *Pedicularis alaschanica* | *Pedicularis* | Orobanchaceae |
| *Pedicularis gruina* | *Pedicularis* | Orobanchaceae |
| *Pedicularis kansuensis* | *Pedicularis* | Orobanchaceae |
| *Pedicularis oederi* | *Pedicularis* | Orobanchaceae |
| *Pedicularis plicata* | *Pedicularis* | Orobanchaceae |
| *Meconopsis horridula* | *Meconopsis* | Papaveraceae |
| *Lancea tibetica* | *Lancea* | Phrymaceae |
| *Lagotis brachystachya* | *Lagotis* | Plantaginaceae |
| *Veronica ciliata* | *Veronica* | Plantaginaceae |
| *Agropyron cristatum* | *Agropyron* | Poaceae |
| *Agrostis filipes* | *Agrostis* | Poaceae |
| *Calamagrostis epigeios* | *Calamagrostis* | Poaceae |
| *Elymus atratus* | *Elymus* | Poaceae |
| *Elymus burchan-buddae* | *Elymus* | Poaceae |
| *Eragrostis nigra* | *Eragrostis* | Poaceae |
| *Festuca coelestis* | *Festuca* | Poaceae |
| *Festuca ovina* | *Festuca* | Poaceae |
| *Festuca sinensis* | *Festuca* | Poaceae |
| *Leymus secalinus* | *Leymus* | Poaceae |
| *Littledalea tibetica* | *Littledalea* | Poaceae |
| *Microchloa kunthii* | *Microchloa* | Poaceae |
| *Orinus thoroldii* | *Orinus* | Poaceae |
| *Pennisetum flaccidum* | *Pennisetum* | Poaceae |
| *Poa glauca* | *Poa* | Poaceae |
| *Puccinellia tenuiflora* | *Puccinellia* | Poaceae |
| *Stipa hookeri* | *Stipa* | Poaceae |
| *Stipa purpurea* | *Stipa* | Poaceae |
| *Tripogon filiformis* | *Tripogon* | Poaceae |
| *Trisetum spicatum* | *Trisetum* | Poaceae |
| *Polygala crotalarioides* | *Polygala* | Polygalaceae |
| *Persicaria nepalensis* | *Persicaria* | Polygonaceae |
| *Persicaria vivipara* | *Persicaria* | Polygonaceae |
| *Polygonum macrophyllum* | *Polygonum* | Polygonaceae |
| *Polygonum paleaceum* | *Polygonum* | Polygonaceae |
| *Rheum alexandrae* | *Rheum* | Polygonaceae |
| *Androsace alaschanica* | *Androsace* | Primulaceae |
| *Androsace erecta* | *Androsace* | Primulaceae |
| *Androsace tapete* | *Androsace* | Primulaceae |
| *Lysimachia maritima* | *Lysimachia* | Primulaceae |
| *Adonis coerulea* | *Adonis* | Ranunculaceae |
| *Anemone demissa* | *Anemone* | Ranunculaceae |
| *Aquilegia rockii* | *Aquilegia* | Ranunculaceae |
| *Callianthemum pimpinelloides* | *Callianthemum* | Ranunculaceae |
| *Delphinium caeruleum* | *Delphinium* | Ranunculaceae |
| *Ranunculus longicaulis* | *Ranunculus* | Ranunculaceae |
| *Ranunculus potaninii* | *Ranunculus* | Ranunculaceae |
| *Thalictrum alpinum* | *Thalictrum* | Ranunculaceae |
| *Thalictrum squamiferum* | *Thalictrum* | Ranunculaceae |
| *Trollius farreri* | *Trollius* | Ranunculaceae |
| *Alchemilla glabra* | *Alchemilla* | Rosaceae |
| *Cotoneaster affinis* | *Cotoneaster* | Rosaceae |
| *Dasiphora fruticosa* | *Dasiphora* | Rosaceae |
| *Geum aleppicum* | *Geum* | Rosaceae |
| *Potentilla anserina* | *Potentilla* | Rosaceae |
| *Potentilla argyrophylla* | *Potentilla* | Rosaceae |
| *Potentilla bifurca* | *Potentilla* | Rosaceae |
| *Potentilla discolor* | *Potentilla* | Rosaceae |
| *Potentilla multifida* | *Potentilla* | Rosaceae |
| *Potentilla plumosa* | *Potentilla* | Rosaceae |
| *Potentilla xizangensis* | *Potentilla* | Rosaceae |
| *Sibbaldia cuneata* | *Sibbaldia* | Rosaceae |
| *Sibbaldia procumbens* | *Sibbaldia* | Rosaceae |
| *Saxifraga tibetica* | *Saxifraga* | Saxifragaceae |
| *Oreosolen wattii* | *Oreosolen* | Scrophulariaceae |
| *Stellera chamaejasme* | *Stellera* | Thymelaeaceae |
| *Viola biflora* | *Viola* | Violaceae |

**Table S3** Effects of abiotic variables on plot-level plant diversities (i.e., taxonomic, functional, and Faith’s PD) at the transect scale (i.e., northern and southern sites). Site was treated as a random effect. The corresponding *P*-value and marginal *R^2^m* (variance explained by fixed effects only) are displayed. Response variables were log-transformed for normality.

| ***Northern sites*** | | | | | | | | |
| --- | --- | --- | --- | --- | --- | --- | --- | --- |
|  | **Taxonomic richness** | |  | **Functional richness** | |  | **Faith's PD** | |
| Variable | *P-value* | *R^2^m* |  | *P-value* | *R^2^m* |  | *P-value* | *R^2^m* |
| Longitude | **0.038** | **0.287** |  | 0.197 | 0.052 |  | 0.209 | 0.112 |
| Latitude | 0.152 | 0.152 |  | 0.176 | 0.057 |  | 0.283 | 0.083 |
| MAP | 0.120 | 0.176 |  | 0.214 | 0.048 |  | 0.418 | 0.049 |
| MAT | 0.330 | 0.074 |  | 0.143 | 0.067 |  | 0.196 | 0.118 |
| SAP | 0.697 | 0.012 |  | 0.942 | 0.001 |  | 0.606 | 0.020 |
| STC | 0.883 | 0.002 |  | 0.682 | 0.006 |  | 0.759 | 0.017 |
| SCN | 0.297 | 0.084 |  | 0.495 | 0.017 |  | 0.383 | 0.056 |
| ***Southern sites*** | | | | | | | | |
|  | **Taxonomic richness** | |  | **Functional richness** | |  | **Faith's PD** | |
| Variable | *P-value* | *R^2^m* |  | *P-value* | *R^2^m* |  | *P-value* | *R^2^m* |
| Longitude | **0.009** | **0.335** |  | **0.007** | **0.313** |  | **0.021** | **0.276** |
| Latitude | 0.225 | 0.081 |  | 0.230 | 0.071 |  | 0.516 | 0.024 |
| MAP | **0.016** | **0.290** |  | **0.005** | **0.326** |  | **0.022** | **0.270** |
| MAT | 0.195 | 0.095 |  | 0.479 | 0.026 |  | 0.265 | 0.072 |
| SAP | 0.789 | 0.004 |  | 0.717 | 0.006 |  | 0.912 | 0.001 |
| STC | **0.009** | **0.330** |  | **0.048** | **0.185** |  | **0.040** | **0.224** |
| SCN | 0.548 | 0.021 |  | 0.945 | 0.001 |  | 0.716 | 0.008 |

MAP: Mean annual temperature; MAT: Mean annual precipitation; SAP: Soil available phosphorus content; STC: Soil total carbon content; SCN: Soil carbon/nitrogen ratio.

**Table S4** Effects of abiotic variables on the plot-level weighted mean value of plant traits at the regional (i.e., all sites) and transect scale (i.e., northern and southern sites). The site was treated as a random effect. The corresponding *P-value* and marginal *R^2^m* (variance explained by the fixed effects only) are displayed.

| ***Regional scale (all sites)*** | | | | | | | | | | | | | | | | | |
| --- | --- | --- | --- | --- | --- | --- | --- | --- | --- | --- | --- | --- | --- | --- | --- | --- | --- |
|  | **CWM.LC** | |  | **CWM.LN** | |  | **CWM.LP** | |  | **CWM.LCN** | |  | **CWM.H** | |  | **CWM.SLA** | |
| Variable | *P-value* | *R^2^m* |  | *P-value* | *R^2^m* |  | *P-value* | *R^2^m* |  | *P-value* | *R^2^m* |  | *P-value* | *R^2^m* |  | *P-value* | *R^2^m* |
| Longitude | **0.006** | **0.204** |  | 0.1 | 0.088 |  | 0.755 | 0.003 |  | **<0.001** | **0.285** |  | 0.755 | 0.003 |  | 0.292 | 0.033 |
| Latitude | 0.316 | 0.031 |  | 0.155 | 0.067 |  | 0.957 | 0.001 |  | 0.535 | 0.012 |  | 0.957 | 0.001 |  | 0.588 | 0.009 |
| MAP | **<0.001** | **0.354** |  | 0.984 | 0.001 |  | 0.312 | 0.028 |  | **<0.001** | **0.353** |  | 0.312 | 0.028 |  | **0.024** | **0.14** |
| MAT | 0.185 | 0.053 |  | **0.018** | **0.176** |  | 0.823 | 0.001 |  | 0.498 | 0.014 |  | 0.823 | 0.001 |  | 0.516 | 0.013 |
| SAP | 0.61 | 0.008 |  | 0.834 | 0.001 |  | 0.505 | 0.012 |  | 0.662 | 0.002 |  | 0.505 | 0.012 |  | 0.742 | 0.003 |
| STC | 0.276 | 0.036 |  | 0.062 | 0.111 |  | 0.405 | 0.019 |  | 0.057 | 0.103 |  | 0.405 | 0.019 |  | 0.385 | 0.022 |
| SCN | 0.563 | 0.01 |  | 0.76 | 0.003 |  | 0.671 | 0.005 |  | 0.594 | 0.009 |  | 0.671 | 0.005 |  | 0.536 | 0.012 |
| ***Transect scale (northern sites)*** | | | | | | | | | | | | | | | | | |
|  | **CWM.LC** | |  | **CWM.LN** | |  | **CWM.LP** | |  | **CWM.LCN** | |  | **CWM.H** | |  | **CWM.SLA** | |
| Variable | *P-value* | *R^2^m* |  | *P-value* | *R^2^m* |  | *P-value* | *R^2^m* |  | *P-value* | *R^2^m* |  | *P-value* | *R^2^m* |  | *P-value* | *R^2^m* |
| Longitude | 0.918 | 0.001 |  | 0.902 | 0.001 |  | 0.711 | 0.009 |  | 0.857 | 0.002 |  | 0.711 | 0.008 |  | 0.687 | 0.009 |
| Latitude | 0.442 | 0.026 |  | 0.393 | 0.033 |  | 0.759 | 0.006 |  | 0.258 | 0.060 |  | 0.759 | 0.006 |  | 0.790 | 0.004 |
| MAP | 0.447 | 0.025 |  | 0.850 | 0.002 |  | 0.623 | 0.014 |  | 0.521 | 0.020 |  | 0.623 | 0.014 |  | 0.677 | 0.010 |
| MAT | 0.294 | 0.047 |  | 0.084 | 0.119 |  | 0.720 | 0.008 |  | **0.018** | **0.213** |  | 0.720 | 0.008 |  | 0.682 | 0.009 |
| SAP | 0.098 | 0.108 |  | 0.387 | 0.033 |  | 0.697 | 0.009 |  | 0.111 | 0.112 |  | 0.697 | 0.009 |  | 0.576 | 0.017 |
| STC | 0.925 | 0.001 |  | 0.815 | 0.003 |  | 0.396 | 0.041 |  | 0.811 | 0.003 |  | 0.396 | 0.041 |  | 0.396 | 0.039 |
| SCN | 0.584 | 0.013 |  | 0.647 | 0.010 |  | 0.580 | 0.018 |  | 0.595 | 0.014 |  | 0.580 | 0.018 |  | 0.600 | 0.015 |
| ***Transect scale (southern sites)*** | | | | | | | | | | | | | | | | | |
|  | **CWM.LC** | |  | **CWM.LN** | |  | **CWM.LP** | |  | **CWM.LCN** | |  | **CWM.H** | |  | **CWM.SLA** | |
| Variable | *P-value* | *R^2^m* |  | *P-value* | *R^2^m* |  | *P-value* | *R^2^m* |  | *P-value* | *R^2^m* |  | *P-value* | *R^2^m* |  | *P-value* | *R^2^m* |
| Longitude | **0.021** | **0.247** |  | 0.232 | 0.084 |  | 0.733 | 0.006 |  | **0.005** | **0.337** |  | 0.733 | 0.006 |  | 0.314 | 0.057 |
| Latitude | 0.984 | 0.001 |  | 0.189 | 0.096 |  | 0.157 | 0.099 |  | 0.586 | 0.015 |  | 0.157 | 0.099 |  | 0.351 | 0.047 |
| MAP | **0.002** | **0.378** |  | 0.655 | 0.012 |  | 0.457 | 0.029 |  | **0.002** | **0.396** |  | 0.457 | 0.029 |  | 0.088 | 0.153 |
| MAT | 0.652 | 0.011 |  | **0.013** | **0.313** |  | 0.099 | 0.136 |  | 0.727 | 0.007 |  | 0.099 | 0.136 |  | 0.460 | 0.031 |
| SAP | 0.904 | 0.001 |  | 0.842 | 0.002 |  | **0.037** | **0.183** |  | 0.855 | 0.002 |  | **0.037** | **0.183** |  | 0.096 | 0.127 |
| STC | 0.531 | 0.021 |  | 0.069 | 0.182 |  | 0.484 | 0.026 |  | 0.184 | 0.090 |  | 0.484 | 0.026 |  | 0.569 | 0.018 |
| SCN | 0.621 | 0.013 |  | 0.667 | 0.011 |  | 0.637 | 0.012 |  | 0.693 | 0.008 |  | 0.637 | 0.012 |  | 0.930 | 0.001 |

CWM.LC: Community weighted mean of leaf carbon content; CWM.LN: Community weighted mean of leaf nitrogen content; CWM.LP: Community weighted mean of leaf available phosphorus content; CWM.LCN: Community weighted mean of leaf carbon/nitrogen ratio; CWM.H: Community weighted mean of plant height; CWM.SLA: Community weighted mean of specific leaf area; MAP: Mean annual temperature; MAT: Mean annual precipitation; SAP: Soil available phosphorus content; STC: Soil total carbon content; SCN: Soil carbon/nitrogen ratio.

**Table S5** Effects of abiotic variables on plot-level functional and phylogenetic structure at the regional (i.e., all sites) and transect scales (i.e., northern and southern sites). Site was treated as a random effect. The corresponding *P-value*, marginal *R^2^m* (variance explained by the fixed effects only) and conditional *R^2^c* (variance explained by both the fixed and random effects) are displayed.

|  | ***Regional scale (all sites)*** | | | |  | ***Transect scale (northern sites)*** | | | |  | ***Transect scale (southern sites)*** | | | |
| --- | --- | --- | --- | --- | --- | --- | --- | --- | --- | --- | --- | --- | --- | --- |
|  | **SES.MFD** | | **SED.MPD** | |  | **SES.MFD** | | **SED.MPD** | |  | **SES.MFD** | | **SED.MPD** | |
| Variable | *P-value* | *R^2^m* | *P-value* | *R^2^m* |  | *P-value* | *R^2^m* | *P-value* | *R^2^m* |  | *P-value* | *R^2^m* | *P-value* | *R^2^m* |
| Longitude | 0.784 | 0.002 | 0.864 | 0.001 |  | 0.636 | 0.007 | 0.112 | 0.077 |  | 0.673 | 0.009 | 0.726 | 0.005 |
| Latitude | **0.027** | **0.098** | 0.328 | 0.016 |  | 0.591 | 0.009 | 0.080 | 0.093 |  | 0.300 | 0.051 | 0.879 | 0.001 |
| MAP | **0.022** | **0.104** | 0.561 | 0.006 |  | 0.846 | 0.001 | 0.100 | 0.082 |  | 0.133 | 0.108 | 0.897 | 0.001 |
| MAT | 0.073 | 0.066 | **0.032** | **0.073** |  | 0.524 | 0.013 | 0.052 | 0.113 |  | 0.767 | 0.004 | 0.055 | 0.121 |
| SAP | 0.438 | 0.013 | 0.137 | 0.036 |  | 0.228 | 0.045 | 0.451 | 0.018 |  | 0.325 | 0.042 | 0.171 | 0.059 |
| STC | 0.854 | 0.001 | 0.578 | 0.001 |  | 0.773 | 0.003 | 0.945 | 0.001 |  | 0.566 | 0.017 | 0.746 | 0.004 |
| SCN | 0.387 | 0.016 | **0.033** | **0.072** |  | 0.649 | 0.007 | 0.148 | 0.064 |  | 0.171 | 0.091 | **0.024** | **0.161** |

MAP: Mean annual temperature; MAT: Mean annual precipitation; SAP: Soil available phosphorus content; STC: Soil total carbon content; SCN: Soil carbon/nitrogen ratio; SES.MFD: standardized effect size of mean functional trait distance; SES.MPD: standardized effect size of mean pairwise phylogenetic distance.

**Figure S1** Correlation between phylogenetic diversity indices calculated from phylogenetic trees constructed based on V. PhyloMaker and DNA sequences.


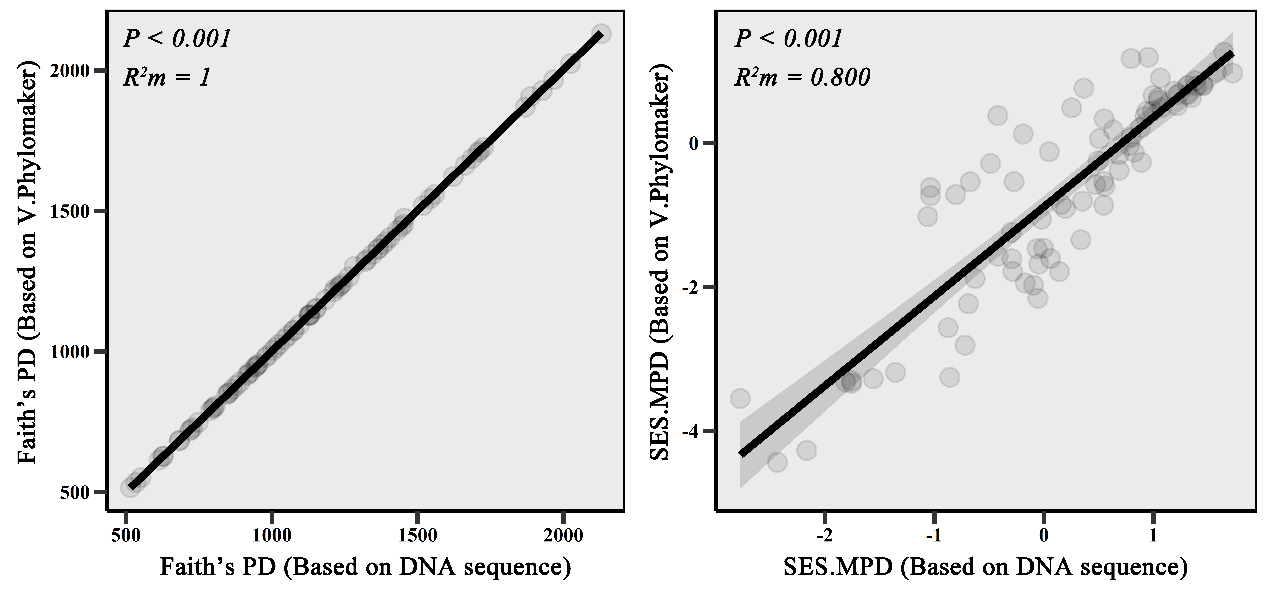


**Figure S2** Pearson’s correlations between abiotic variables.
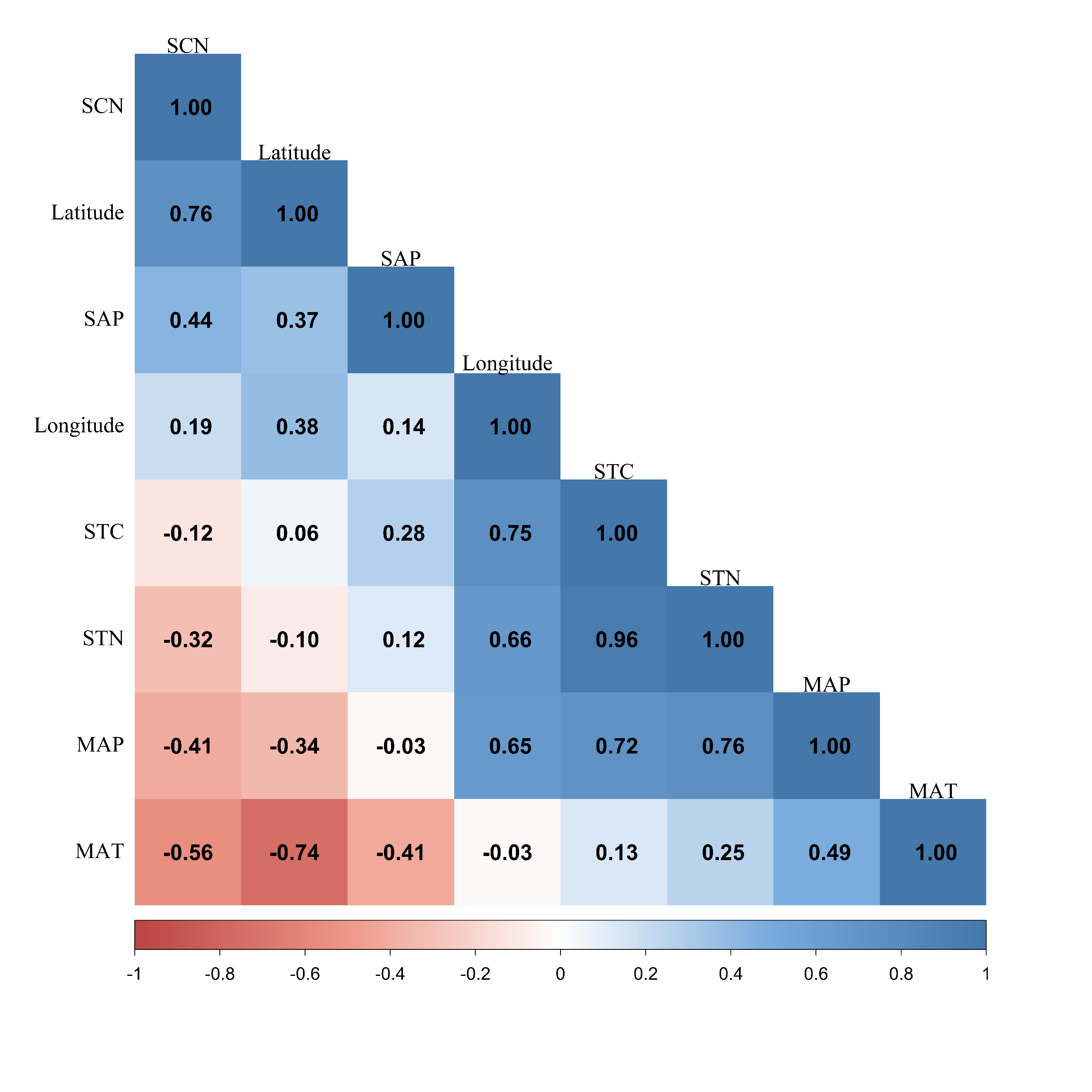


**Figure S3** Fraction of the variation in plant taxonomic (a), functional (b), and phylogenetic (c) diversities explained by spatial and environmental predictors at the transect scale (i.e., northern and southern sites). Significance: ^#^*P < 0.1*. MAP: Mean annual temperature; MAT: Mean annual precipitation; SAP: Soil available phosphorus content; STC: Soil total carbon content; SCN: Soil carbon/nitrogen ratio.


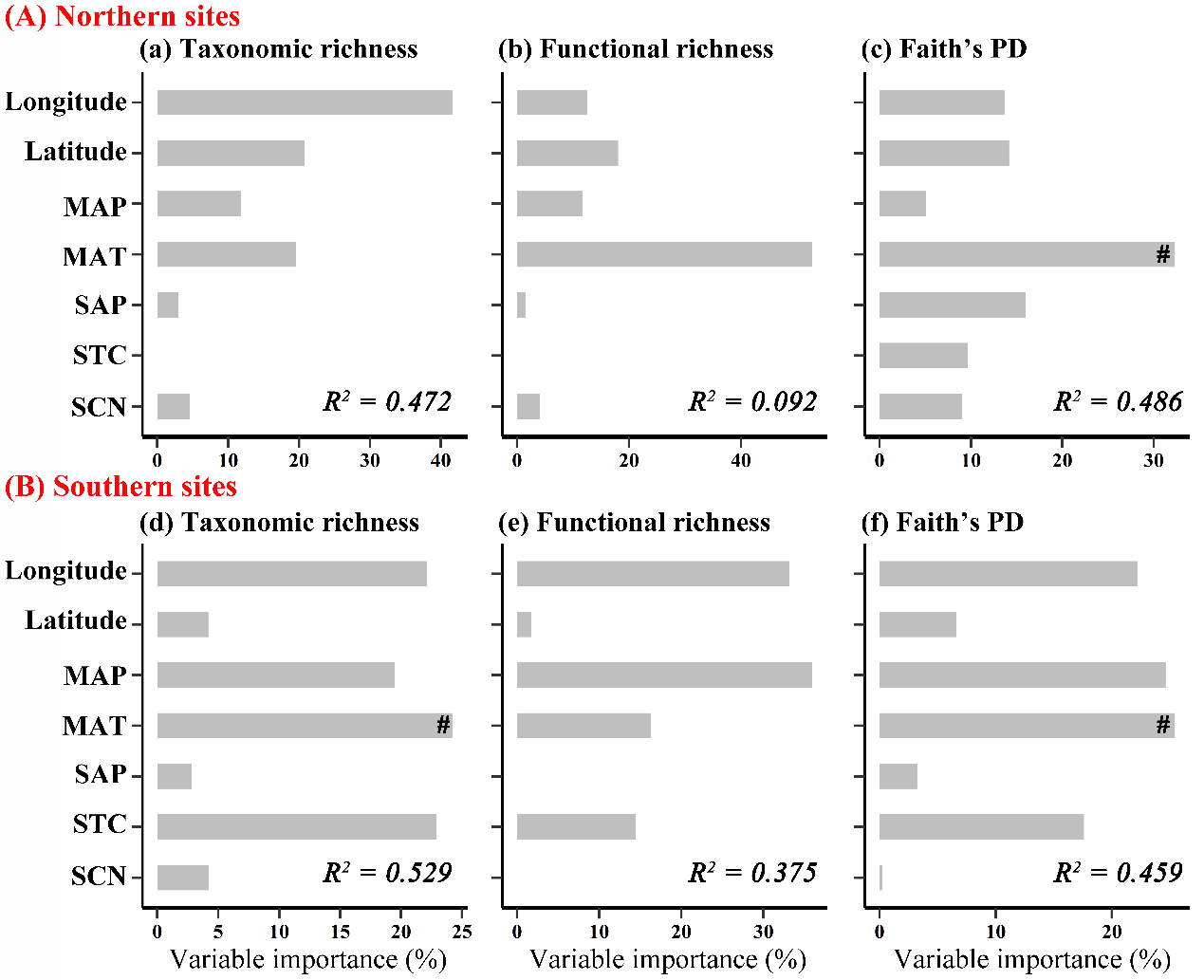


**Figure S4** Fraction of the variation in standardized effect size of mean pairwise phylogenetic distance (SES.MPD) of plant community explained by spatial and environmental predictors at the region (i.e., all sites) and transect scale (i.e., northern and southern sites). Significance: ^#^*P < 0.1*, ^*^*P < 0.05*, ^**^*P < 0.01*, ^***^*P < 0.001*. MAP: Mean annual temperature; MAT: Mean annual precipitation; SAP: Soil available phosphorus content; STC: Soil total carbon content; SCN: Soil carbon/nitrogen ratio. The SES.MPD was calculated based on the phylogenetic tree constructed by the V.PhyloMaker.


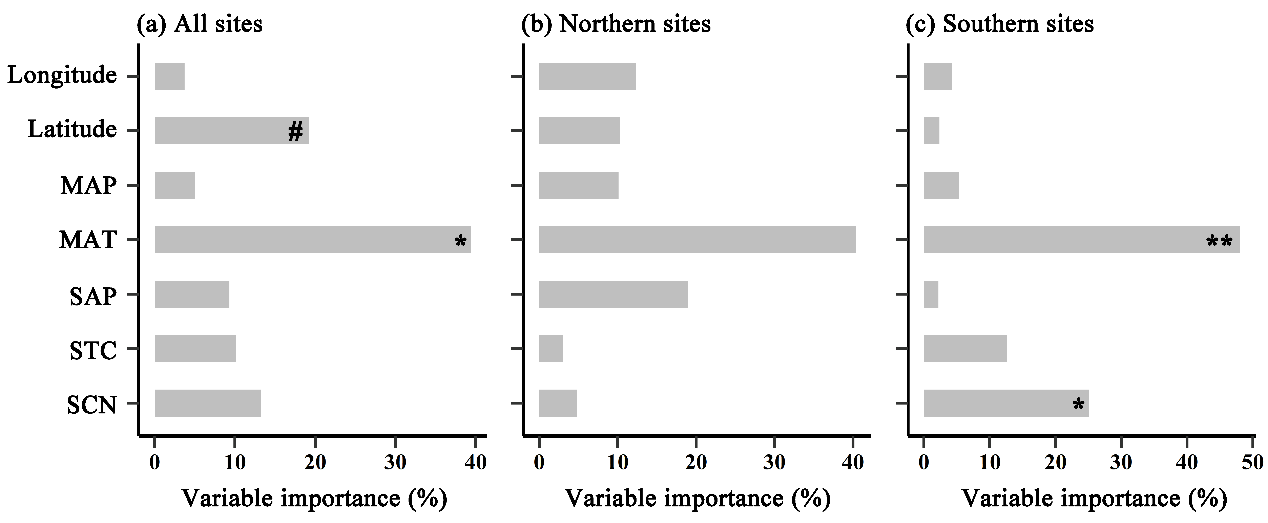


**Figure S5** Fraction of the variation in plant community structure and the weighted mean values of plant traits explained by spatial and environmental predictors at the transect scale (i.e., northern and southern sites). Significance: ^#^*P < 0.1*, ^*^*P < 0.05*, ^**^*P < 0.01*, ^***^*P < 0.001*. CWM.LC: Community weighted mean of leaf carbon content; CWM.LN: Community weighted mean of leaf nitrogen content; CWM.LP: Community weighted mean of leaf available phosphorus content; CWM.LCN: Community weighted mean of leaf carbon/nitrogen ratio; CWM.H: Community weighted mean of plant height; CWM.SLA: Community weighted mean of specific leaf area. MAP: Mean annual temperature; MAT: Mean annual precipitation; SAP: Soil available phosphorus content; STC: Soil total carbon content; SCN: Soil carbon/nitrogen ratio; SES.MFD: standardized effect size of mean functional trait distance; SES.MPD: standardized effect size of mean pairwise phylogenetic distance.


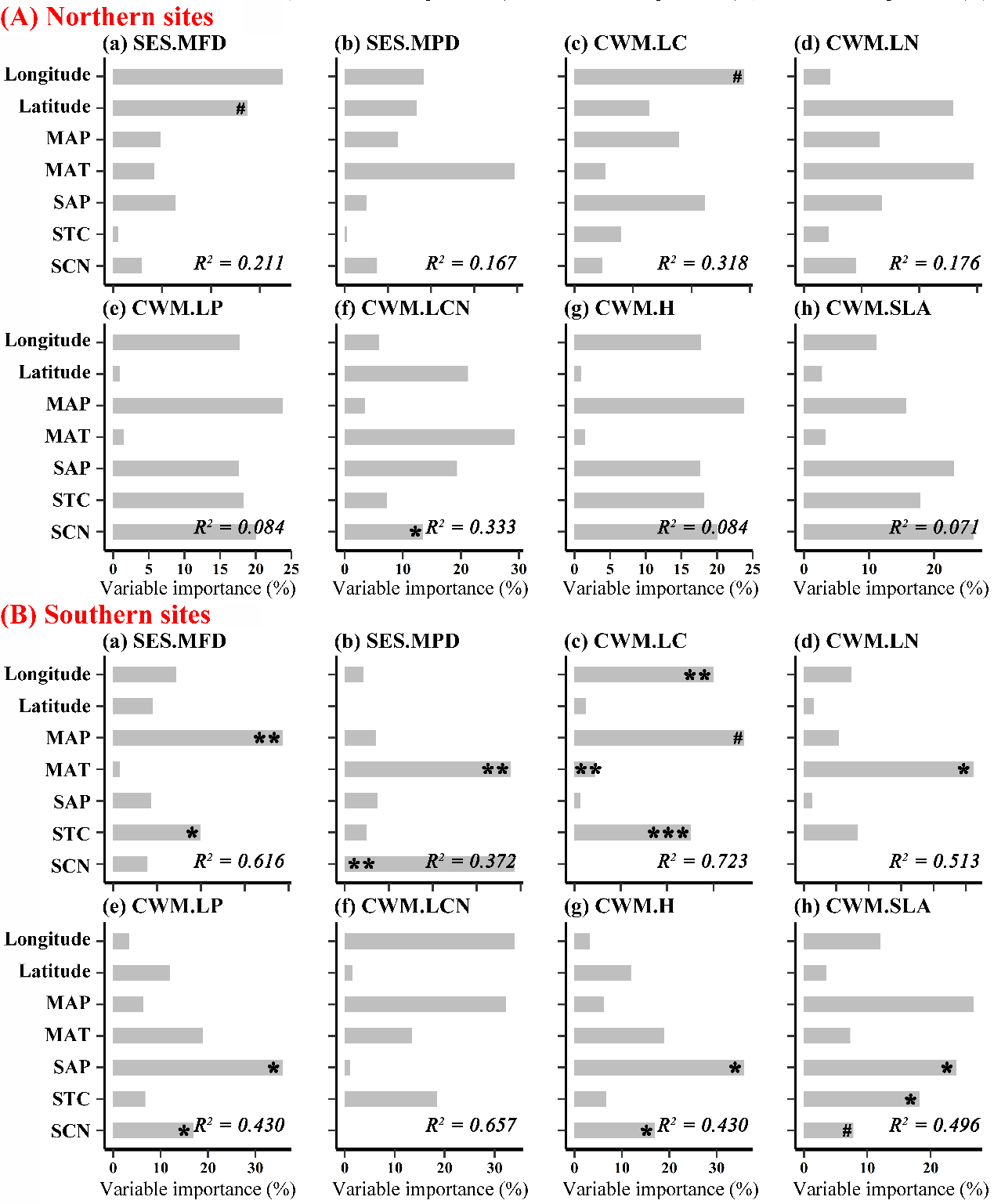


**Figure S6** Fraction of the variation in species composition explained by the spatial (i.e., SPAT) and environmental (i.e., ENV) variables, and their interaction (i.e., ENV * SPA) at the transect scale (i.e., northern and southern sites).


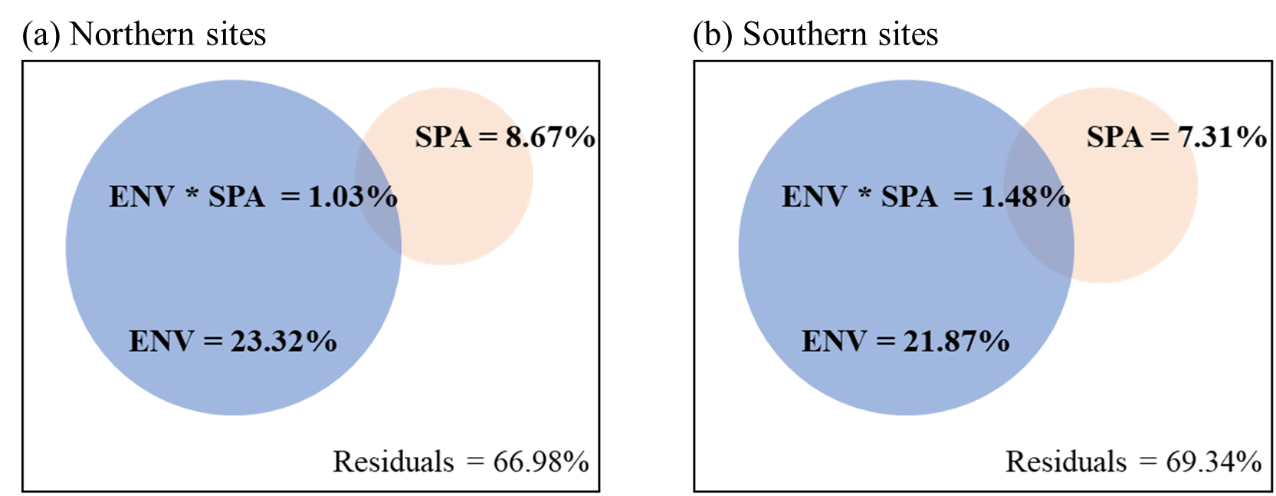

Supplement: Supplementary file 1 — Data S1. [file ECE3-15-e71599-s001.docx]
